# Developing an automated risk management tool to minimize bird and bat mortality at wind facilities

**DOI:** 10.1007/s13280-015-0707-z

**Published:** 2015-10-27

**Authors:** Julia Robinson Willmott, Greg M. Forcey, Lauren A. Hooton

**Affiliations:** Normandeau Associates, Inc., 102 NE 10 Avenue, Gainesville, FL 32601 USA

**Keywords:** Monitoring, Migration, Collision, ATOM, Risk, Offshore

## Abstract

A scarcity of baseline data is a significant barrier to understanding and mitigating potential impacts of offshore development on birds and bats. Difficult and sometimes unpredictable conditions coupled with high expense make gathering such data a challenge. The Acoustic and Thermographic Offshore Monitoring (ATOM) system combines thermal imaging with acoustic and ultrasound sensors to continuously monitor bird and bat abundance, flight height, direction, and speed. ATOM’s development and potential capabilities are discussed, and illustrated using onshore and offshore test data obtained over 16 months in the eastern USA. Offshore deployment demonstrated birds tending to fly into winds and activity declining sharply in winds >10 km h^−1^. Passerines showed distinct seasonal changes in flight bearing and flew higher than non-passerines. ATOM data could be used to automatically shut down wind turbines to minimize collision mortality while simultaneously providing information for modeling activity in relation to weather and season.

**Electronic supplementary material:**

The online version of this article (doi:10.1007/s13280-015-0707-z) contains supplementary material, which is available to authorized users.

## Introduction

Current developments and future plans to make extensive use of onshore and offshore wind resources in both Europe and the USA (MMS 2006; DOE 2008; EEA 2009) have increased awareness that wind turbines have the potential to adversely impact birds and bats (e.g., Drewitt and Langston 2006; Arnett et al. 2008; Cryan 2011). However, because significant portions of bird and bat migration occur at night (Kunz et al. 2007), directly monitoring the timing and magnitude of migration is difficult and costly, particularly in offshore environments (Normandeau Associates 2012).

Most regulatory agencies across Europe and the USA recommend that wind energy developers assess potential impacts to birds and bats, among other wildlife groups, from wind turbines. These impacts may be direct, such as mortality from collisions, or indirect, such as loss of foraging and breeding habitat (Kunz et al. 2007; USFWS 2012). Thus, data are required on daily and monthly use of proposed development sites by individual species, particularly flight height and direction, given the critical importance of these data for collision risk modeling (e.g., Krijgsveld et al. 2005; Barclay et al. 2007). However, because of logistical and financial limitations, risk assessment studies of bats and migratory birds at planned wind farms are routinely based on non-continuous surveys at discrete times of the year (Cook et al. 2012).

Recent advances in technologies such as radar and thermal imaging allow quantification of some aspects of bird and bat migration (e.g., Hill et al. 2014; Horton et al. 2015), and recording and analysis of distinctive vocalizations from active migratory flights can provide species-specific information at a given place and time (Horton et al. 2015). While acoustic detection alone can be a powerful tool, acoustic detectors are only effective if birds and bats are emitting calls. Bats may not always emit echolocation calls when flying through large open areas (Kunz et al. 2007), and some bird species are not inherently vocal (e.g., shearwaters; del Hoyo et al. 1992). Acoustic sensors allow for qualification of some of the bird and bat movements, which, when combined with visual sensors, would also allow for some quantification.

Normandeau Associates Inc. with assistance from the U.S. Department of the Interior, Bureau of Ocean Energy Management, and Cornell Lab of Ornithology (CLO) designed and tested the Acoustic and Thermographic Offshore Monitoring (ATOM) system—a combination of thermal imaging and acoustic and ultrasound sensors—to continuously survey bird and bat species potentially affected by onshore and offshore wind development. ATOM was tested during onshore and offshore deployments to examine the feasibility of the system, in terms of design, ease of deployment and maintenance, ability to gather data, and to inform future improvements. In this paper, we describe the development of the ATOM system, consider how it might be used for real-time impact mitigation in operational wind farms and for gathering appropriate data to assess and minimize impacts of proposed new developments, and discuss how it could contribute to addressing crucial gaps in knowledge of bird and bat ecology. To illustrate this, we present two test datasets and show how ATOM data may be used to address key questions such as whether there are predictable differences in bird and bat abundance, flight direction, and flight height in response to time of day and season. Answers to these questions in near real time could be used to mitigate and minimize collision mortality. We therefore discuss how, based on our data, the system might be modified to collect, monitor, and evaluate information to automatically inform shutdown and reduce collision risk at wind farms.

## Materials and methods

### ATOM system

**Fig. 1:**
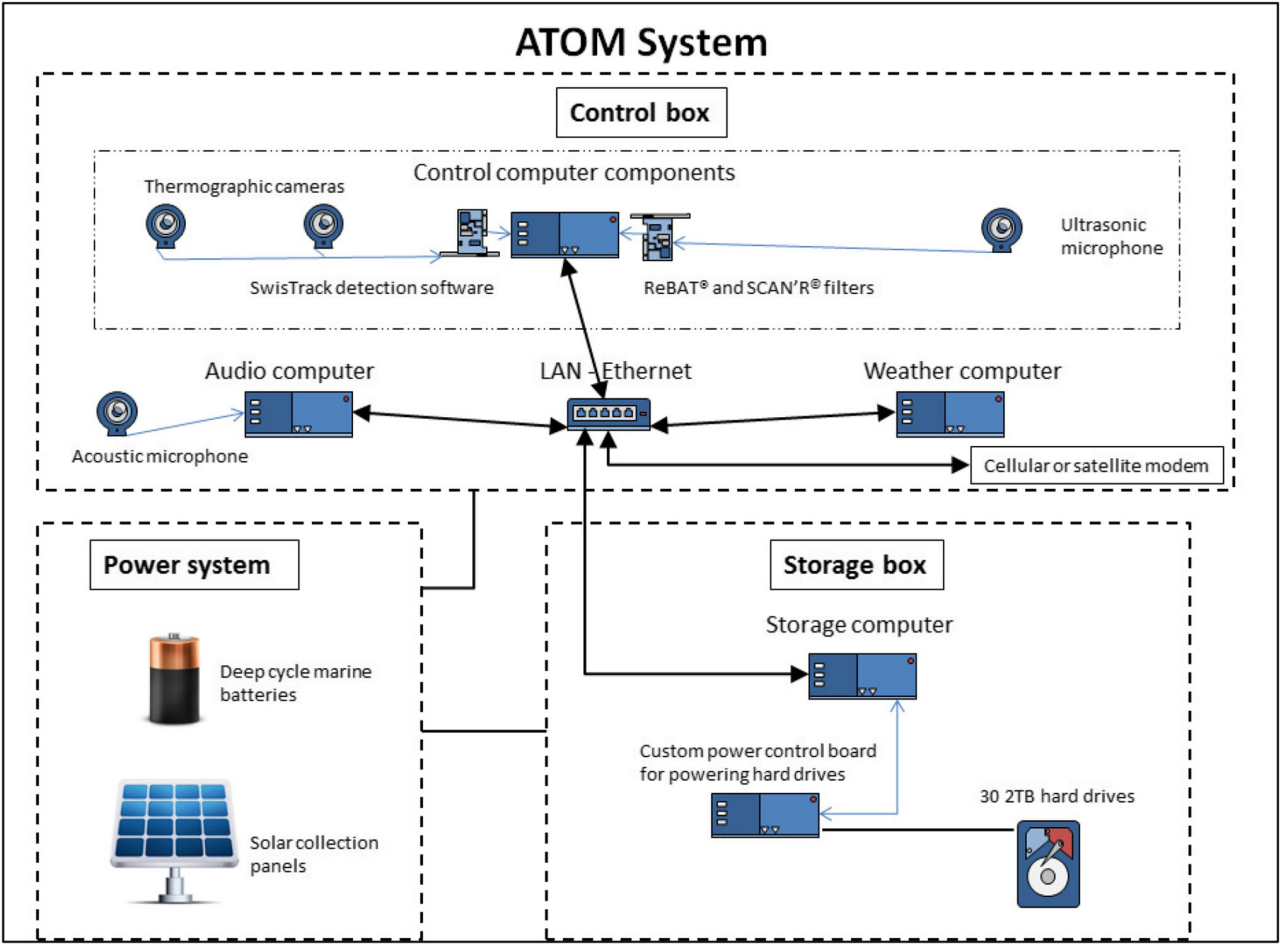
Composition of the central system control and communication elements of the fully integrated ATOM system. ATOM has four data collection sensors: thermographic camera, audio acoustic, ultrasound acoustic, and weather. Blue lines with arrows represent components of the computer, such as boards required to control the cameras, ultrasonic microphone, and the custom power control board that powers the storage hard drives. The power system is connected to both the control system (top box) and the storage system (bottom right box) and represented as a black line without arrows. Black lines with arrows represent ethernet communication connections between the autonomous computers

The ATOM system used for monitoring bats and migratory birds in this study included a combination of deployable thermographic, acoustic, and ultrasound sensors that autonomously record data and transmit them to a central site for storage and analysis. System software includes algorithms and protocols for managing and analyzing large volumes of data recorded by the sensors. The system is described fully by Normandeau Associates (2014) and, in its final deployment within this study, included the following: a Verizon™ cellular modem and a Hughes^®^ satellite modem connected to different computers; two FLIR Tau 320 (Forward Looking Infrared) cameras and an integrated custom-built wiper system; two Bolide Technology Group BT-MP8087 acoustic microphones; one AR-125 ultrasonic microphone (Binary Acoustic Technology, Tucson); an integrated meteorological system recording visibility, temperature, wind speed and direction, and humidity (Columbia Weather Systems MicroServer); and a power monitoring system (Power Control Hub) with built-in satellite communication (see Fig. 1). The solar system consisted of solar collection panels; deep-cycle, sealed lead acid marine batteries; and charge controllers. The audio computer had bidirectional communication between the nodes and the host module using a LAN-based Ethernet connection. All sensor data were received by the control computer and transferred to the storage system. The five separate computers that comprised the central core of the ATOM system were housed in two, custom-fabricated weatherproof containers: one for the storage computer, including 32 storage drives (30 × 2 TB, 2 × 3 TB), and one for the other four computers and the two thermographic cameras (see Fig. 1).

The system’s two thermal cameras look up from the main control computer box through thermally transparent germanium ‘windows’ covering the holes on each end of a metal bar. The windows on the upper surface of the bar were covered by movable metal covers with rubber O-rings that cleaned the windows as needed by applying fluid to the upper surface of the windows and then moving the O-rings across the surface, much like a windscreen wiper.

The power monitoring system remotely monitored the overall health and functionality of the system by reporting the following: voltage draw of each component; operating state; input and output voltages; input and output currents of the solar charge controller; input voltages to the power control board; the temperature of numerous system components including the control computer, solar charge controller, power control board, storage computer box, and hard drives; and the internal relative humidity of the control and storage boxes. It also reported the number of system restarts for various system computers, the amount of hard drive space available and used on the storage and control computers, and the network bandwidth used. Internal logging constantly monitored system health and assisted in identifying timing and causes of any malfunction and indications of system weakness, allowing targeted maintenance.

### ATOM system deployment

**Fig. 2:**
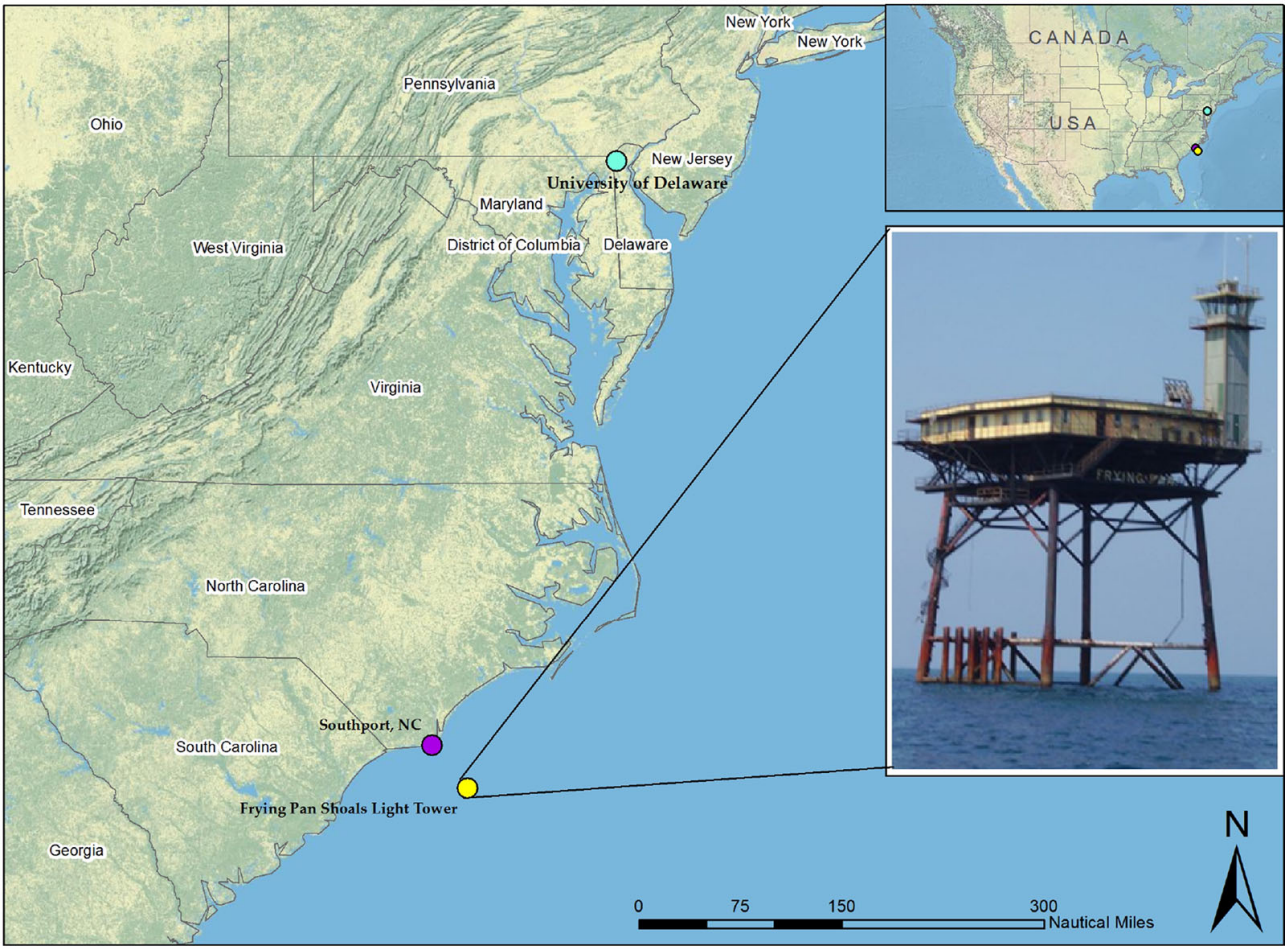
Location of University of Delaware-Lewes, the terrestrial deployment location at an operating wind turbine, and Frying Pan Shoals Light Tower, the offshore deployment on the platform shown on the lower image (inset). These coastal and offshore ATOM deployment sites are located in Delaware and North Carolina, USA, and their locations within continental USA shown on the upper image (inset)

The overall functioning of the system and its ability to record target species was tested during an installation beneath the terrestrial wind turbine at University of Delaware-Lewes (UD-Lewes; 38°46′58.53″N, 75°9′53.41″W) from 18 July 2011 to 9 August 2011 (Table 1; Fig. 2). Eight bat species are resident in Delaware (Supplementary material, Table S1), and all were expected to be active at UD-Lewes during the time of our testing (DNREC 2012). After this short terrestrial test, the system was deployed on Frying Pan Shoals Light Tower (FPSLT) for 15 months (December 2011 to March 2013), the maximum amount of time that funding allowed. FPSLT is an 80-ft platform constructed in 1966 that is located 29 miles offshore southeast of Southport, North Carolina (33°29′N, 77°35′W; Fig. 2). This location is far enough from shore that many true pelagic taxa can be found including storm-petrels, shearwaters, jaegers, and albatrosses. Other taxa, such as gulls and terns that typically inhabit both near shore and pelagic environments could also be expected. During spring and fall migration, non-pelagic taxa, such as neotropical passerines, were also anticipated to pass through (Poole 2005), although much remains unknown about migration strategies of non-pelagic species so far offshore because of the difficulties associated with collecting such data. Offshore bat activity is not well documented, and patterns of activity and species abundances offshore remain unclear. Species that are sometimes reported offshore in this region, and thus potentially detectable at FPSLT, are the migratory eastern red bat (*Lasiurus borealis*), hoary bat (*L. cinereus*), and silver-haired bat (*Lasionycteris noctivagans*), but a few others have also been documented offshore (Pelletier et al. 2013; Peterson et al. 2014; Table S1).

**Table 1:** Recording effort (hours) summarized by month, location, and time of day for each ATOM system sensor component at two test deployment locations in the eastern USA. Delaware is an onshore location; Frying Pan is 29 miles offshore (see Fig. 2 for their geographical location)

Month and year	Location	Recording hours								
		Thermographic			Acoustic			Ultrasonic		
		Total	Diurnal	Nocturnal	Total	Diurnal	Nocturnal	Total	Diurnal	Nocturnal
Jul 2011	Delaware	**77**	47	30				**51**	24	27
Aug 2011	Delaware	**1277**	73	55				**69**	39	31
Dec 2011	Frying Pan	**127**	54	73	**153**	67	87	**518**	251	267
Jan 2012	Frying Pan	**12**	3	9	**31**	13	17	**570**	298	272
Feb 2012	Frying Pan									
Mar 2012	Frying Pan							**337**	201	135
Apr 2012	Frying Pan	**490**	255	236	**661**	341	319	**689**	370	320
May 2012	Frying Pan	**285**	165	120	**494**	286	208	**539**	309	229
Jun 2012	Frying Pan	**583**	351	233	**585**	352	233			
Jul 2012	Frying Pan	**148**	90	58	**154**	88	67			
Aug 2012	Frying Pan	**171**	107	65						
Sep 2012	Frying Pan	**558**	305	254	**406**	225	181			
Oct 2012	Frying Pan	**442**	220	222	**474**	241	233			
Nov 2012	Frying Pan				**356**	178	179			
Dec 2012	Frying Pan				**96**	41	55			

Bold numbers represent the TOTAL numbers = nocturnal and diurnal data added

Data transfer, storage, and analysis occurred at Normandeau’s Gainesville office in Florida. Data were uploaded to the ATOM-dedicated Linux server from the hard drives in the ATOM data storage system. Ultrasound data files were collected and stored as 205 kHz, 16 bit PCM ‘wav’ files, and thermographic data in a proprietary PSIR format. Acoustic audio files were originally recorded as DAT files, subsequently converted to CAF files for storage, and eventually analyzed as 16 bit PCM ‘wav’ files. Near real-time data downloading has since been developed for use when cellular connectivity is available, but during the deployment reported here, the hard drives were collected approximately every 3 months depending on weather-related accessibility. Drives containing copies of acoustic data were forwarded to CLO for additional analysis.

#### Thermographic analysis

**Fig. 3:**
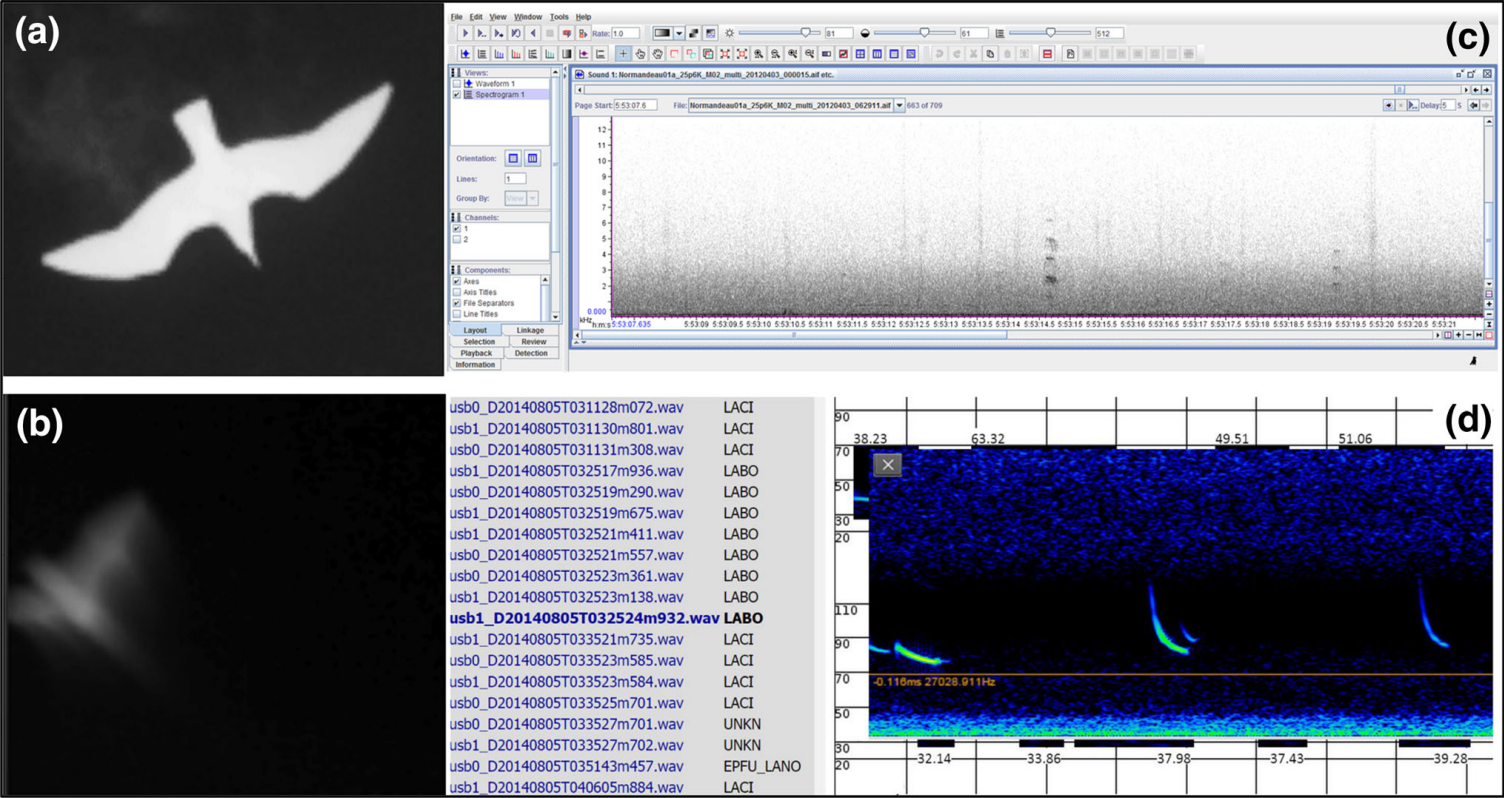
Sensor output collected by ATOM during deployment, showing examples of (a) thermographic bird and (b) thermographic bat images, (c) a spectrogram of bird calls, and (d) an example of a SonoBat display of a bat pass with the automated species classification displayed. These thermographic images show examples in which the distinction between birds and bats can be made confidently based on the animal’s shape in the image

Thermographic data collected from UD-Lewes by the ATOM system underwent manual review restricted to those data within timestamps identified during ultrasound data analysis as containing targets. Automated and manual quality control reviews were completed on all thermographic video data collected from FPSLT between December 2011 and October 2012. Data were processed through an automated target detection program named SwisTrack (see Normandeau Associates 2014), which produced video segments (tracks) of potential targets. This filter was adjusted to eliminate tracking of all turbine blades at the terrestrial deployment and most clouds and insects at both deployments. Distance, velocity, and bearing of objects were estimated by triangulating the coordinates of the objects from each of two cameras. Distance was corrected for height above sea level by adding the distance of the platform and camera from the ocean. This was a correction of 32 m; birds could not be recorded under this altitude and thus all observations occurred within the range of altitudes defined as the rotor swept zone of marine turbines (20–200 m). Velocity and bearing were calculated by measuring the change in distance over time among frames. In instances where the object was recorded across multiple frames, the median distance of the object from the camera was reported. The accuracy and error of the calculations were characterized in field tests using targets of known size, distance, direction, and speed. Flight trajectories of foraging bats deviate rapidly and unpredictably from a straight line, whereas the flight paths of birds tend to be straighter (Kunz et al. 2007). It has also been suggested that some bats may use relatively straight flight trajectories while migrating, and other bats may have overall tendencies toward straighter flight trajectories (Ghose et al. 2006; Kunz et al. 2007); therefore, straighter flight trajectories were classified as bird/bat and not used in bird or bat analyses if no other evidence was available for distinguishing them. In some cases with low flying animals, the shape of the animal was distinctive enough to manually identify whether it was a bird or bat (see Fig. 3). Size of the object, assessed by distance from camera, was also used as a distinguishing feature. Raw video segments were manually reviewed to quality control the automated detection performance.

#### Ultrasound acoustic analysis

The full-spectrum ultrasound acoustic data were analyzed using automated and manual processes developed for use with the ReBAT^®^ (Remote Bat Acoustic Technology) system. ReBAT was developed for bat acoustic monitoring at wind energy facilities; specifically, it allows long-term acoustic monitoring from meteorological towers and/or where the blade attaches to the turbine (the nacelle) so that bats can be detected within the rotor swept zone, where fatalities occur. The ultrasound acoustic systems were fully online and constantly monitored (via cell or satellite networks) for functionality, and when deployed within cellular range at UD-Lewes, recorded bat data were sent to offsite servers for storage and analysis. When deployed at FPSLT, data were stored onto hard drives and collected with all other data. As ultrasonic data were recorded, they were automatically (automated target detection) filtered by SCAN’R© filtering software (Binary Acoustic Technology, Tucson, AZ) to remove noise files. This program recognizes a potential bat pass event and produces a 1.7-s duration ‘wav’ file; any time at least two consecutive potential bat echolocation calls are recorded. SCAN’R uses the ultrasound spectrographic patterns of bat calls to recognize potential bat calls (Binary Acoustic Technology 2010). Once filtered by the SCAN’R software, remaining files were run through an additional ReBAT.com filter to remove noise files not captured by SCAN’R. Additionally, a subset of the files removed by the ReBAT.com filter was manually reviewed to ensure that no bat calls were being discarded as noise. The remaining bat calls were manually identified to species or species group using expert knowledge as well as SonoBat™ 3 (Joe Szewczak; Arcata, CA), an acoustic identification software program that was periodically used to obtain a second opinion regarding species ID for calls that had call parameters potentially assigned to more than one species. Manual bat call identification involved viewing spectrograms and assessing certain parameters of the echolocation calls; specifically, minimum and maximum frequency, call duration, and inter-pulse interval. These parameters were then compared to known values for bat species found within the monitoring area (Fenton and Bell 1981). Each 1.7-s file usually only contained one pass (sequence of ≥2 bat calls). Occasionally, a file contained more than one bat pass belonging to the same or different species. On the rare occasion that this occurred, the file was still counted as one pass.

#### Audio acoustic analysis

Analyses of migrant songbirds from the FPSLT deployment focused on nocturnal flight calls. Nocturnal flight calls are species-specific vocalizations of up to several syllables that generally are in the 1–11 kHz frequency band and 50–300 ms in duration. These calls are the primary vocalizations given by many species of birds during long, sustained flights characteristic of nocturnal migration (Evans and O’Brien 2002). Raven Pro Sound Analysis Software v.1.5 (Bioacoustics Research Program, Cornell Lab of Ornithology 2013) was used to process and analyze the sound recordings using two different Band Limited Energy Detectors to detect possible nocturnal flight calls in two discrete frequency ranges: a high range encompassing 6000–11 000 Hz to capture sparrows and warbler calls and a lower range between 2250 and 3750 Hz to capture calls of thrushes, shorebirds, and other bird species. To reduce the potentially high number of false detections, a Random Forest model (Liaw and Wiener 2002) was used to rank the likelihood that a given detection was an actual flight call. For this analysis, acoustic analysts manually reviewed the tens of thousands of ranked candidate call detections and confirmed each as true calls or noise. All true calls were annotated to the most specific taxonomic level possible by experts in bird call identification.

#### Analysis of relationship between environmental factors and bird and bat activity

Detection ability was determined by reviewing 10 % of the images manually for targets and comparing this number with those detected from automated analysis. To permit comparisons across species, times of day, and seasons, automated thermographic data were corrected for both detection ability and survey time. Variation in animal distribution and density across time of day and across season can impact turbine avoidance behavior and collision risk. Consequently, the ability to predict activity on a daily and seasonal level can help provide suitable collision mitigation strategies.

Detection success values were calculated on a monthly basis. Detections were corrected by survey time by assuming the same number of targets occurred during times when the thermographic camera was not running as when it was running. Corrections for survey time were performed across each analysis period: day, night, and all hours. Corrected abundance (*A*_c_) was calculated by summing the number of birds across each month (*A*_0_), dividing this by the automated detection correction for the given month (*S*_s_), and dividing the outcome of this division by the proportion of the month that was surveyed (*O*_t_). Corrected abundance was thus calculated according to the following:

$$ A_{c} = \frac{{\frac{{A_{o} }}{{S_{s} }}}}{{O_{t} }}. $$

Like detection success corrections, abundance corrections were performed on a monthly basis so that the timeframe was wide enough for a large enough sample size. In addition to evaluating abundance data from automated analyses, comparisons of flight altitude, flight bearing, and flight velocity were also examined by season. Results are illustrated for all birds combined, and separate results are also presented differentiating behavioral patterns for passerines and non-passerines. These behavioral patterns include flight altitude, bearing, and velocity, relevant due to differences in life history characteristics between passerines and other species. These metrics were chosen because they directly influence collision risk and can be used to inform smart shutdown of wind turbines. Birds <20 cm in size were classified as passerines and birds > 30 cm as non-passerines. Birds between 20 and 30 cm were not included in this categorization because of overlap in the sizes of some passerines with some Laridae species. Relationships associated with potential risk of collision, between weather variables (including wind speed and wind direction) and abundance, flight altitude, and flight direction were evaluated by examining scatterplots of the data and drawing qualitative conclusions. Statistical significance was evaluated by comparing the 95 % confidence intervals among different groups. Groups whose 95 % confidence intervals did not overlap were considered significantly different from each other (*α* = 0.05).

## Results

### ATOM system deployment and performance

During its operation, the ATOM system gathered thermographic, audible acoustic, and ultrasound acoustic data to monitor bird and bat activity (Fig. 3). The primary purpose of the UD-Lewes deployment was to test the functionality of the thermographic and acoustic systems. As ultrasound acoustic detection software was already available, ultrasound data were fully analyzed from all data collected at UD-Lewes. Targeted review was performed on thermographic data, thereby selecting those data points for which time stamps corresponded with automated ultrasound detections. This was done to assess if species-specific activity data could be determined.

Although data were gathered through a very wide range of weather conditions and during day and night, data collection was not continuous. A number of issues caused system malfunctions when deployed on FPSLT causing periods when one or more sensors did not gather data. For the thermographic data, the target detection program SwisTrack initially produced 10 065 video segments, or tracks, of potential targets. At UD-Lewes, 8.6% of thermographic data were identified as bird/bat. At FPSLT, only birds were identified and no animals were identified as bat or as bird/bat.

### Bats

Ultrasonic data could only be collected at FPSLT from 6 December 2011 through 28 May 2012 because the microphone became damaged due to the harsh marine environment and stopped recording. This prevents strong conclusions as to the presence or absence, with any regularity, of bats occurring later than May at FPSLT, although some passes would have been expected in both the thermographic and ultrasound data should bats have been migrating through this area from wintering habitats. However, the ultrasonic microphone on the ATOM system was able to successfully record bat echolocation calls at the UD-Lewes terrestrial turbine over multiple nights and in various weather conditions and did not appear to be significantly hindered by the noise of the turbine.

**Fig. 4:**
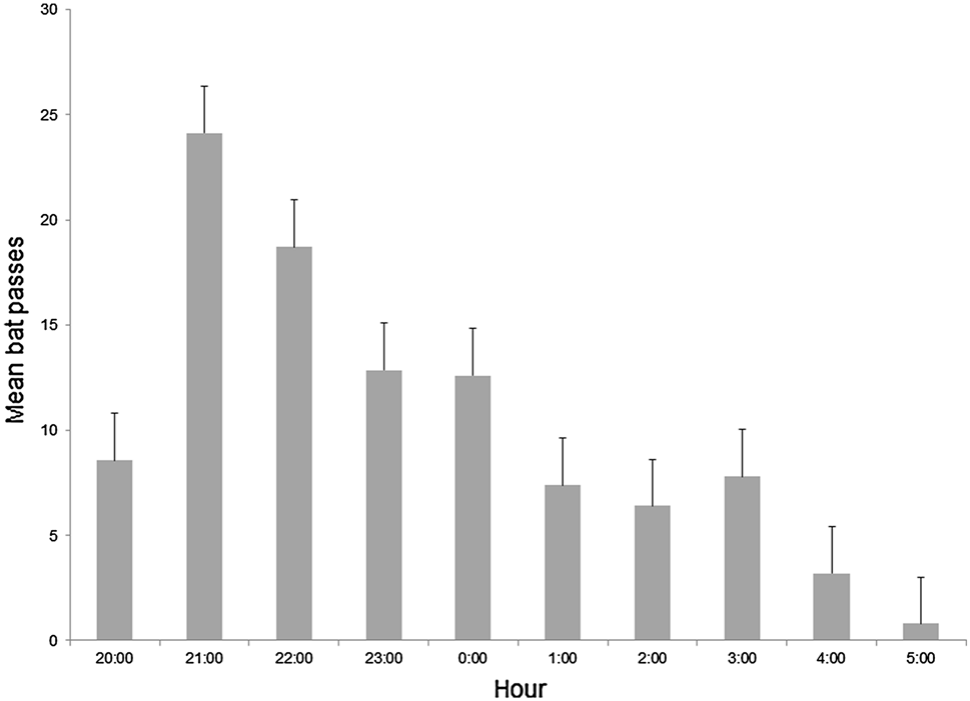
Average number of bat passes (± standard error) per hour recorded by the ultrasound detector during ATOM system deployment at UD-Lewes (July–August 2011). Null data are omitted from this figure. The time of sunset during this deployment was at approximately 20:10 and the time of sunrise was at approximately 6:00. No bats were recorded flying before 20:00 and after 6:00

From the seven nights of data collected at UD-Lewes, 641 acoustic ultrasound bat passes were detected and identified. Most of the passes could be confidently identified to five species, with the remainder lumped into species groups. Of the calls that could be identified to species, eastern red bats were detected most often (44% of identified individuals), followed by big brown bats and silver-haired bats (Table 2). Overall bat activity was greatest within the first few hours following sunset, after which activity waned for the remainder of the night (Fig. 4).

**Table 2:** Bat species detected during ATOM system test deployment at the University of Delaware-Lewes (July–August 2011); data presented in taxonomic order

Common name	Scientific name	Total passes
Eastern red bat	*Lasiurus borealis*	180
Hoary bat	*Lasiurus cinereus*	14
Silver-haired bat	*Lasionycteris noctivagans*	75
Big brown bat	*Eptesicus fuscus*	121
Tri-colored bat	*Perimyotis subflavus*	18
	Unidentified to species	233
Total number of passes		641

**Fig. 5:**
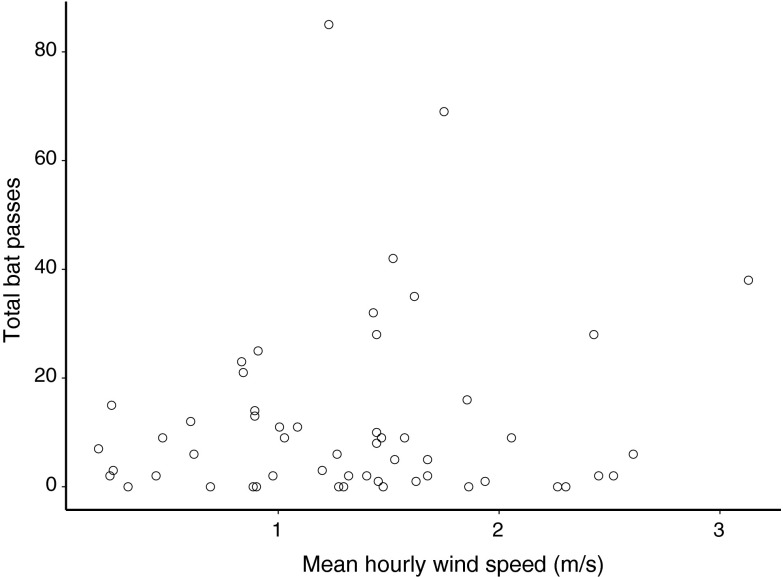
Bat passes with average wind speed for each nocturnal hour that the ultrasonic detector was operational during the ATOM system deployment at UD-Lewes (July–August 2011). Nocturnal hours are defined as after 20:00 and before 06:00

There were 15 thermographic bat detections discovered in the data associated with ultrasound detections in the UD-Lewes analysis (Fig. 3; Table S2). Two of these detections occurred at the same moment ultrasonic bat detections were reported and were considered matches (i.e., same individual bat recorded on both ultrasound and thermographic sensors), and both proved to be flying below the rotor swept area. The identities of these bats were unambiguously determined from the ultrasound recordings as eastern red bat (altitude 43.4 m above ground level [agl], mean bearing [flight direction] of 6.73 NNE) and big brown bat (altitude 41.9 m agl, mean bearing of 12.02 NNE, velocity 6.4 m s^−1^) (see shaded rows in Table S2). The altitudinal range for bats captured in thermographic imagery was 18 m to 73 m. Most bats flew at >40 m (*n* = 6) and within the rotor swept area, and 86 % of thermographic bat passes were heading in a NNE direction (see Table S2). Although wind speed only varied from 0 to 4 m s^−1^ during the nocturnal hours of operation, bat activity was highest when wind speeds were between 0.5 and 2.5 m s^−1^ (Fig. 5), which is below the wind speed threshold (3.5 m s^−1^) at which many commercial wind turbines become operational (begin activity spinning and generating energy).

### Birds

All of the bird data discussed here came from the main offshore deployment at FPSLT.

#### Thermographic data

Birds were visible in 1763 video segments. During manual review, it was found that 237 tracks identified by SwisTrack concerned flight paths of birds circling above the cameras, in and out of the field of view. Further manual inspection of 10 % of unfiltered and randomly selected monthly recording hours showed SwisTrack success rates of bird detections from within the video imagery ranging from below 15 % to over 60 %. Success varied depending on the number of video frames that contained multiple birds. It became evident that SwisTrack was not always able to discriminate between individual birds when multiple birds were flying within the camera’s view at the same time. The review of unfiltered data resulted in 246 birds being recorded. The final number of individual birds detected flying over the thermal cameras at FPSLT was 1492.

#### Audio acoustic data

A total of 2640 calls were recorded from 39 taxonomic units detected in files identified for analysis by software targeting calls typically used during migration. These represented at least 34 manually identified migratory species (Table 3). An additional seven species were manually identified in remaining data identified for analysis by software targeting files containing multiple vocalizations by gulls and terns. These were laughing gull (*Leucophaeus atricilla*), ring-billed gull (*Larus delawarensis*), herring gull (*L. argentatus*), common tern (*Sterna hirundo*), Forster’s tern (*S. forsteri*), royal tern (*Thalasseus maximus*), and sandwich tern (*T. sandvicensis*).

**Table 3:** Call counts of all bird species (presented in taxonomic order) detected by nocturnal flight call analyses and manually identified to species or taxonomic group. Data were collected at FPSLT deployment (03 April–12 December 2012) during spring (03 April–31 May), breeding season (01 June–15 July), fall (16 July–31 October), and winter (01 November–12 December)

Common Name	Scientific Name	Spring	Breeding	Fall	Winter	Total
Species level identifications						
Royal tern	*Thalasseus maximus*	1	0	0	0	1
Least bittern	*Ixobrychus exilis*	0	0	1	0	1
Green heron	*Butorides virescens*	0	0	7	0	7
Veery	*Catharus fuscescens*	0	0	14	0	14
Gray-cheeked thrush	*Catharus minimus*	0	0	81	0	81
Swainson’s thrush	*Catharus ustulatus*	0	0	114	0	114
Hermit thrush	*Catharus guttatus*	0	0	0	20	20
Wood thrush	*Hylocichla mustelina*	1	0	4	0	5
American pipit	*Anthus rubescens*	0	0	0	5	5
Ovenbird	*Seiurus aurocapilla*	13	0	76	0	89
Northern waterthrush	*Parkesia noveboracensis*	0	0	9	0	9
Black-and-white warbler	*Mniotilta varia*	0	0	33	0	33
Prothonotary warbler	*Protonotaria citrea*	1	0	0	0	1
Common yellowthroat	*Geothlypis trichas*	12	0	21	0	33
American redstart	*Setophaga ruticilla*	0	0	69	0	69
Cape May warbler	*Setophaga tigrina*	0	0	476	0	476
Northern parula	*Setophaga americana*	3	0	209	0	212
Magnolia warbler	*Setophaga magnolia*	0	0	6	0	6
Bay-breasted warbler	*Setophaga castanea*	0	0	14	0	14
Blackburnian warbler	*Setophaga fusca*	0	0	4	0	4
Yellow warbler	*Setophaga petechia*	2	0	2	0	4
Chestnut-sided warbler	*Setophaga pensylvanica*	0	0	2	0	2
Blackpoll warbler	*Setophaga striata*	16	0	32	0	48
Black-throated blue warbler	*Setophaga caerulescens*	1	0	54	0	55
Palm warbler	*Setophaga palmarum*	1	0	324	0	325
Yellow-rumped warbler	*Setophaga coronata*	7	0	196	0	203
Canada warbler	*Cardellina canadensis*	2	0	0	0	2
Chipping sparrow	*Spizella passerina*	18	0	0	0	18
Savannah sparrow	*Passerculus sandwichensis*	0	0	10	0	10
White-throated sparrow	*Zonotrichia albicollis*	24	0	1	0	25
Dark-eyed junco	*Junco hyemalis*	1	0	0	0	1
Blue grosbeak	*Passerina caerulea*	0	0	1	0	1
Indigo bunting	*Passerina cyanea*	30	0	9	0	39
Bobolink	*Dolichonyx oryzivorus*	0	0	11	0	11
Genus-level identifications						
Thrush sp.	*Catharus* sp.	0	0	29	0	29
Setophaga wood warbler sp.	*Setophaga* sp.	18	0	134	0	152
Family-level identifications						
Species belonging to wood warblers family	*Parulidae* sp.	4	0	20	2	26
Species belonging to buntings family	*Emberizidae* sp.	0	0	3	0	3
Order-level identifications						
Species belonging to the order of passerines	*Passeriformes*	43	3	431	7	484
Class-level identifications						
Birds	*Aves*	6	1	1	0	8
Total		204	4	2398	34	*2640*

#### Data analysis

**Fig. 6:**
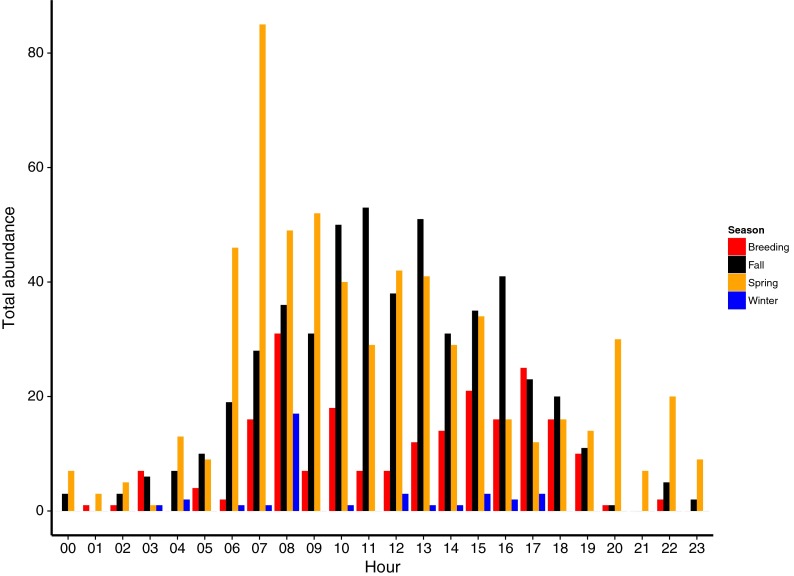
Total bird abundance across all species by season on an hourly basis (local time), based on thermographic data

Seasonal patterns of bird activity are important considerations when modeling potential risk to species or species groups. Audio acoustic analysis showed nocturnal migrant peak call encounters to occur during the fall migratory period with predictably few encounters in the breeding season (*n* = 4) and in winter (*n* = 34; Table 3). Hermit thrush was the most frequently encountered species during winter (*n* = 20; Table 3). For other species, in this case mainly gulls and terns, encounters were common across all seasons. Analyses of thermographic data for all birds including gulls, terns, and frigatebird showed the majority of detections occurring during daylight hours, primarily between 6 am and 6 pm, with much lower activity detected at night (Fig. 6). At this location, day length has only small fluctuations across all seasons, and the same trend in daytime activity occurred consistently throughout the year with twice as many daytime detections occurring in all seasons. Over the course of the ATOM system monitoring period, activity peaked in spring and fall with lower activity reported during summer and winter (Fig. 6). Hourly abundance varied by season with peak spring abundance between 6 and 10 am and peak fall abundance between 10 am and 2 pm (Fig. 6). Migration behavior in April showed higher than usual nocturnal activity, although diurnal activity was consistently higher than nocturnal activity through all months.

**Fig. 7:**
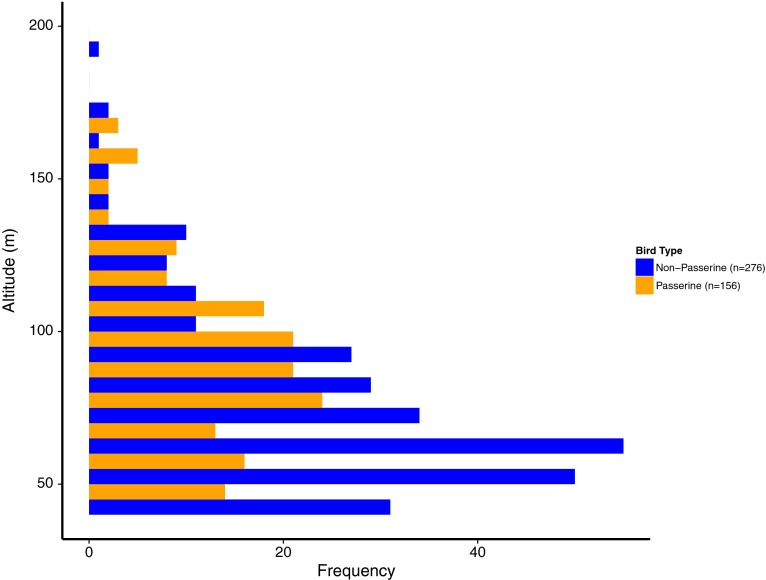
Frequency of flight heights (in meters above sea level) for passerines and non-passerines based on thermographic data. The thermographic detector was at 32 m asl; birds under this height were not recorded. Rotor Swept Zones range between 20 m to 200 m

Flight height, an important dimension when considering collision risk, was consistent throughout the day with slightly greater average heights being detected in the early evening, though the variation around these estimates was high. Throughout the year, average flight altitude was lowest during fall (*x̄* = 83.4 m, SE = 1.4 m) and highest during the spring (*x̄* = 96.4 m, SE = 1.7 m). During the breeding season, a higher mean altitude was observed near sunrise (*x̄* = 158.4 m, SE = 50.9 m), but this difference was not significant due to the large variability around the mean; such a trend was not apparent in other seasons. Winter data were sparse for flight altitude and no clear trends were visible. Passerine activity was limited to high activity during just a few weeks (Table 3). Therefore, for an assessment of collision risk, it is helpful to be able to differentiate passerine flight height from non-passerine flight height, as gulls and tern activity were more evenly distributed. A comparison of flight height frequency of passerines to non-passerines showed that passerine flight altitudes were frequently greater than those of non-passerines (Fig. 7).

**Fig. 8:**
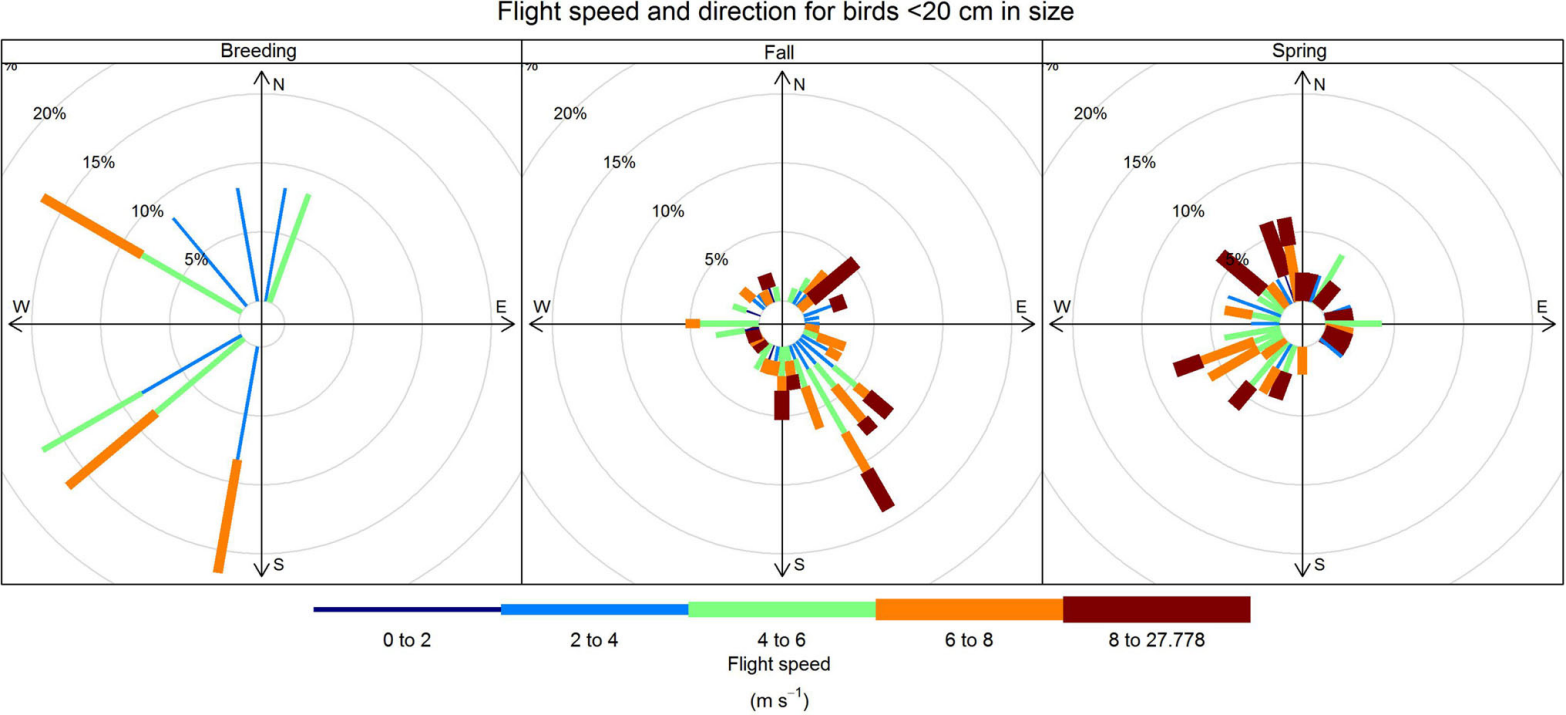
Seasonal variation in bearing and flight speed for passerines recorded throughout the duration of the study. Longer bars indicate greater relative occurrence in a given direction. Mean flight speed is reported in green text for each season. Percent indicates proportion of activity in any given direction and at any given speed

Flight bearing can indicate the direction of a nesting colony or feeding area, important factors when considering breeding colony impacts or feeding displacement impacts from development. Seasonal differences in flight bearing were observed for passerines, but similar trends were not evident with non-passerines. Passerines showed strong tendencies to fly to the south and southeast during the fall and to the northwest during the spring (Fig. 8). Flight bearings for non-passerines did not mirror these trends and no discernable patterns were evident.

**Fig. 9:**
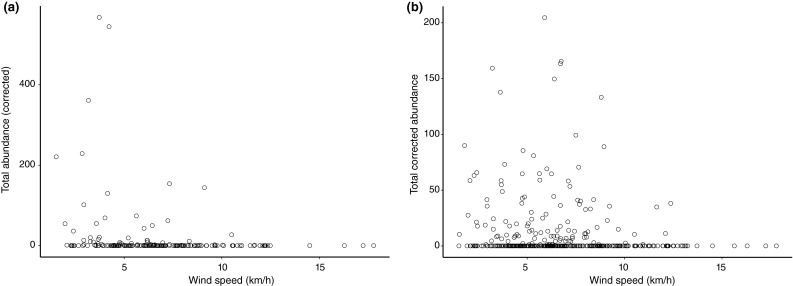
Mean bird abundance of all observations by wind speed for both (a) acoustic and (b) thermographic data showing a sharp decline once wind speeds reach 10 km h^−1^, the average cut-in speed for offshore wind turbines. Corrected abundance accounts for both varying detection success and the amount of time surveyed per month

Weather variables, frequently associated with bird collision risk, were found to be associated with bird abundance, flight altitude, and flight direction. Birds occurred consistently through the range of wind speeds up until around 10 km h^−1^, the average cut-in speed for offshore wind turbines, when abundance declined sharply. Both audible acoustic and thermographic data showed this trend (Fig. 9). There was an indication that fewer birds were flying with cross-winds. There was little relationship between wind speed and flight speed with consistent flight speeds being reported across the range of wind speeds.

## Discussion

Our studies demonstrated that remote surveying of birds and bats at onshore and offshore wind farms is possible using the ATOM system. ATOM was able to gather data on bird and bat abundance, as well as their flight height, speed, and direction of flight, crucial inputs for the modeling of collision risk. Where data on the same individuals were recorded simultaneously by multiple sensors, identification to species level was also possible. With sufficient time and deployment in areas of higher bird or bat activity, the ATOM system could provide unprecedented information about the movement and behavior of individual species. Our data show that ATOM’s video and acoustic ultrasound automated analysis systems would allow for transmission of near real-time activity information should cellular connectivity be available. This ability creates an opportunity to make the system capable of mitigating potential collisions by informing targeted shutdown of wind turbines.

While the deployments brought out clear capabilities, they also highlighted some important areas for improvement. For example, there was high diurnal activity in the offshore environment of non-vocalizing birds that were detected thermographically, but species-specific identification was impossible because of a lack of identified associated acoustic data. Data gathering by the ultrasound sensors during the offshore deployment was restricted by frequent failure due to harsh conditions. The newest version of ATOM has benefitted from these lessons with the addition of an ambient light camera and reconfigured ultrasound microphones for improved data gathering and added robustness in the harsh marine environment. Acoustic data are now collected and stored as ‘wav’ files so near real-time analysis can be implemented without conversion from DAT to CAF and finally into ‘wav’ files. Improvements were also made to the power draw and data storage systems. These improvements involved computer reconfigurations and data compression software to reduce the issues that caused periodic system failure in the harsh offshore environment where maintenance visits were restricted by weather, sea-state conditions, and distance from shore.

The ATOM system was able to provide some new insight into bird migration strategies. For example, bird species recorded at FPSLT predictably comprised many trans-Atlantic migrants that winter in the Caribbean and northern parts of South America (including Amazonia), such as Cape May warbler, black-throated blue warbler, gray-cheeked thrush, blackpoll warbler, and bobolink (Table 3). However, in addition, ATOM collected data on a number of unexpected species that are typically thought not to migrate to locations that would require an offshore passage. These species include American pipit, chipping sparrow, and dark-eyed junco. Regarding some known differences in migration strategy among species, ATOM data could demonstrate differential exposure to risk from marine wind farms. For example, 50 % more Cape May warblers were recorded than palm warblers despite the former having an estimated global population of 7 million (ABC 2012) and the latter 20 million (Rich et al. 2004). Both species breed in the far north of USA and Canada and winter in the West Indies, but palm warbler migrate across a broader front with a smaller part of the population migrating over open sea. Furthermore, the timeframe for exposure to risk could be narrowed down: calls from both of these species peaked in October with few records in late September.

Most of the bat activity during the UD-Lewes deployment was from two species that are common in Delaware: the big brown bat and eastern red bat. Based on the late July/early August timeframe, the bats detected at UD-Lewes may not have begun their fall migration behavior. It is likely that as the season progressed, activity would have been much greater for the three long-distance migrant species: hoary bats, silver-haired bats, and eastern red bats (e.g., Hatch et al. 2013; Peterson et al. 2014). Offshore bat activity in eastern North America is mostly limited to the fall migration period (Peterson et al. 2014). Although the ultrasonic microphone at FPSLT stopped functioning after May 2012, precluding any potential detection during the peak fall migration season, the thermographic cameras also failed to pick up any flying bats. It is therefore most likely that bats were not flying so far offshore rather than the thermographic camera failing to detect bats. As in the bird studies, data gathered from the ATOM deployment at UD-Lewes, where bat detections were frequent, show that the system also has a lot to contribute to furthering knowledge of bat migration strategy and factors affecting their abundance.

The intent in presenting our deployment test data was to illustrate, to both researchers and developers, some of the kinds of questions that might potentially be addressed using this type of innovative system. Despite the limited time for which the system was operational (13 months), data were obtained that allowed predictions about bird movement in relation to both season and weather conditions. Such data, gathered intensively in both space and time, have numerous theoretical and practical applications, as discussed below.

New techniques to reduce bird and bat mortality are currently being developed and applied at wind farms around the world, and one of the most significant advances is using relationships between bird/bat abundance and behavior and weather patterns to inform curtailment decisions. For example, bats are most active during low wind speeds, and wind farms have used this knowledge to raise turbine cut-in speeds and successfully reduce bat mortality (Baerwald et al. 2009; Arnett et al. 2011). Multiple weather variables influence bird and bat activity and mortality, however, and these relationships can be statistically modeled (e.g., Weller and Baldwin 2012). These models can then be used to predict when activity and/or mortality will be highest and, therefore, when turbines should be shut down. These methods have been employed at terrestrial wind farms using a combination of weather data and bat acoustic activity data (Korner-Nievergelt et al. 2013). The ATOM system is unique in the field of offshore monitoring, and its visual and acoustic monitoring for birds and bats could provide invaluable information on species-specific occurrence and activity patterns offshore. By including both thermographic video and acoustic sensors, ATOM allows for the detection of those species and individuals that would not otherwise have been recorded by a single detection method, and automatically relates the density of activity and flight height and direction of travel. For example at FPSLT, automated thermographic detection found frigatebird (flight height 55.8 m asl, bearing 99.4° ENE), which does not vocalize, could be identified from thermographic data alone because of its distinctive size and shape. Deployed at offshore sites, the system could collect data on species’ actual offshore migratory activity and behavior rather than inferred or modeled information based on terrestrial data. Data are currently sparse on the amount of flight activity at rotor swept height, a data gap that ATOM could help address. ATOM is a novel tool for gathering appropriate species-specific data to develop and apply more advanced statistical models at onshore and offshore wind facilities. Its ability to relay density, flight height, and flight direction in near real time will ultimately provide real-time curtailment strategies.

## Electronic supplementary material

Supplementary material 1 (PDF 116 kb)
