# Supplementary material for: Developing an automated risk management tool to minimize bird and bat mortality at wind facilities
Source: Ambio. 2015 Oct 27;44(Suppl 4):557–71. doi: 10.1007/s13280-015-0707-z (PMC4623866; doi:10.1007/s13280-015-0707-z)
Supplement: Supplementary file 1 — Supplementary material 1 (PDF 116 kb) [file 13280_2015_707_MOESM1_ESM.pdf]

AMBIO

Electronic Supplementary Material
This supplementary Material has not been peer reviewed.

Title: **Developing an automated risk management tool to minimize bird and bat mortality at wind facilities**

Authors: Julia Robinson Willmott, Greg M. Forcey, Lauren A. Hooton

SUPPLEMENTARY MATERIAL

Table S1 Bat species of Delaware and North American bat species known to be active offshore in the U.S. Atlantic Region (Pelletier et al. 2013; Peterson et al. 2014).

		Known Location	
Common Name	Scientific Name	Delaware	Offshore U.S. Atlantic Region
Eastern red bat	*Lasiurus borealis*	X	X
Hoary bat	*Lasiurus cinereus*	X	X
Silver-haired bat	*Lasionycteris noctivagans*	X	X
Big brown bat	*Eptesicus fuscus*	X	X
Tri-colored bat	*Perimyotis subflavus*	X	X
Little brown bat	*Myotis lucifugus*	X	
Northern long-eared bat	*Myotis septentrionalis*	X	
Evening bat	*Nycticeius humeralis*	X	
Seminole bat	*Lasiurus seminolus*		X

Table S2 Complete data on bat passes recorded over 4 days by the thermographic video cameras during the ATOM test deployment at UD-Lewes (July–August 2011)

Date (YYYY-MM-DD)	Start Time	Flight Altitude (m)	Mean Bearing (degrees and compass)	Velocity (m s^{-1})
2011-08-13	20:59:28.066	Data Not Available	52.79 ENE	Data Not Available
2011-08-13	21:01:04.561	41.9	12.02 NNE	6.4
2011-08-13	21:11:51.759	54.4	18.26 NNE	8.1
2011-08-13	21:56:31.198	Data Not Available	79.17 ENE	Data Not Available
2011-08-13	22:14:19.231	Data Not Available	40.48 NNE	Data Not Available
2011-08-13	23:04:53.330	18	40.78 NNE	3.3
2011-08-13	23:57:31.330	44	19.95 NNE	6.8
2011-08-14	01:53:39.792	Data Not Available	37.19 NNE	Data Not Available
2011-08-14	01:53:41.231	Data Not Available	9.41 NNE	Data Not Available
2011-08-14	01:53:51.429	44.2	1.74 NNE	7.0
2011-08-14	20:56:51.429	43.4	6.73 NNE	5.4
2011-08-15	01:20:09.330	73.3	5.36 NNE	7.7
2011-08-15	02:09:30.561	Data Not Available	6.32 NNE	Data Not Available
2011-08-15	02:31:32.363	Data Not Available	40.32 NNE	Data Not Available
2011-08-15	02:59:31.759	Data Not Available	40.32 NNE	Data Not Available
